# Metformin inhibits human T-cell leukemia virus type 1 transcription through activation of LKB1 and salt-inducible kinases

**DOI:** 10.1186/1742-4690-11-S1-P112

**Published:** 2014-01-07

**Authors:** Hei-Man V Tang, Wei-Wei Gao, Ching-Ping Chan, Yeung-Tung Siu, Chi-Ming Wong, Kin-Hang Kok, Yick-Pang Ching, Hiroshi Takemori, Dong-Yan Jin

**Affiliations:** 1 Department of Biochemistry, The University of Hong Kong, Pokfulam, Hong Kong; 2 Department of Anatomy, The University of Hong Kong, Pokfulam, Hong Kong; 3 Laboratory of Cell Signaling and Metabolism, National Institute of Biomedical Innovation, Osaka, Japan

## 

Human T-cell leukemia virus type 1 (HTLV-1) causes adult T-cell leukemia (ATL). Specific therapeutic and prophylactic agents are not available. Epidemiological studies have established a correlation between long-term use of the commonly prescribed anti-diabetic drug metformin and a decrease in the incidence of breast and other cancers. Whether metformin might also have anti-HTLV-1 and anti-ATL activity is unclear. In this study we demonstrate an inhibitory effect of metformin on HTLV-1 transcription mediated through the activation of LKB1 tumor suppressor and downstream salt-inducible kinases (SIKs). Treatment of HTLV-1- transformed ATL cells or cells transfected with HTLV-1 molecular clone pX1MT with metformin led to reduction in cell-free virion production and inhibition of cell proliferation. This effect was attributed to the activation of LKB1/SIK1 which compromised Tax expression and HTLV-1 transcription. LKB1 and SIKs function as host restriction factors that counteract Tax activation of HTLV-1 long terminal repeats. Expression of LKB1 and activated SIKs effectively blunted Tax activity in a phosphorylation-dependent manner in LKB1-null HeLa cells, whereas compromising these kinases, but not AMP-dependent protein kinases, augmented Tax function in LKB1-proficient HEK293T cells. Activated LKB1 and SIKs associated with Tax. Enforced expression of LKB1 or SIK1 in pX1MT-transfected cells and HTLV-1-transformed ATL cells repressed proviral transcription, whereas depletion of LKB1 boosted Tax expression. Taken together, our findings revealed a potential therapeutic and prophylactic agent for ATL as well as a new negative regulatory function of LKB1 and SIKs in HTLV-1 transcription. (Supported by HKU7661/08M, HKU7674/12M, HKU1/CRF/11G and SKY-MRF-2011).

